# Visual stimuli induce serotonin release in occipital cortex: A simultaneous positron emission tomography/magnetic resonance imaging study

**DOI:** 10.1002/hbm.25156

**Published:** 2020-08-19

**Authors:** Hanne Demant Hansen, Ulrich Lindberg, Brice Ozenne, Patrick MacDonald Fisher, Annette Johansen, Claus Svarer, Sune Høgild Keller, Adam Espe Hansen, Gitte Moos Knudsen

**Affiliations:** ^1^ Neurobiology Research Unit and NeuroPharm, Copenhagen University Hospital, Rigshospitalet Copenhagen Denmark; ^2^ Martinos Center for Biomedical Imaging, Department of Radiology Massachusetts General Hospital, Massachusetts Massachusetts; ^3^ Department of Clinical Physiology Nuclear Medicine and PET, Rigshospitalet, University of Copenhagen Copenhagen Denmark; ^4^ Department of Public Health, Section of Biostatistics University of Copenhagen Copenhagen K Denmark; ^5^ Faculty of Health and Medical Sciences University of Copenhagen Copenhagen Denmark

**Keywords:** [11C]AZ10419369, 5‐HT, 5‐HT1B receptor, simultaneous PET/MR, visual stimulation

## Abstract

Endogenous serotonin (5‐HT) release can be measured noninvasively using positron emission tomography (PET) imaging in combination with certain serotonergic radiotracers. This allows us to investigate effects of pharmacological and nonpharmacological interventions on brain 5‐HT levels in living humans. Here, we study the neural responses to a visual stimulus using simultaneous PET/MRI. In a cross‐over design, 11 healthy individuals were PET/MRI scanned with the 5‐HT_1B_ receptor radioligand [^11^C]AZ10419369, which is sensitive to changes in endogenous 5‐HT. During the last part of the scan, participants either viewed autobiographical images with positive valence (*n* = 11) or kept their eyes closed (*n* = 7). The visual stimuli increased cerebral blood flow (CBF) in the occipital cortex, as measured with pseudo‐continuous arterial spin labeling. Simultaneously, we found decreased 5‐HT_1B_ receptor binding in the occipital cortex (−3.6 ± 3.6%), indicating synaptic 5‐HT release. Using a linear regression model, we found that the change in 5‐HT_1B_ receptor binding was significantly negatively associated with change in CBF in the occipital cortex (*p* = .004). For the first time, we here demonstrate how cerebral 5‐HT levels change in response to nonpharmacological stimuli in humans, as measured with PET. Our findings more directly support a link between 5‐HT signaling and visual processing and/or visual attention.

## Graphical abstract

Here, we study the neural responses to a visual stimulus using simultaneous positron emission tomography/magnetic resonance imaging. We find a significant association between anincrease in cerebral blood flow (CBF) in the occipital cortex along and a decrease in [11C]AZ10419369 binding, suggesting synaptic serotonin (5‐HT) release. To the best of our knowledge, this is the first time that simultaneous changes in CBF and 5‐HT levels in response to physiological stimuli have been measured.

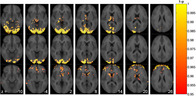

## 1 INTRODUCTION

The central serotonin (5‐HT) system is involved in a large number of functions resulting from its widespread innervation of the entire brain. The neurotransmitter regulates, for example, blood pressure, body temperature, sleep, and feeding behavior (Berger, Gray, & Roth, 2009), but it also plays a role in psychiatric disorders such as anxiety (Craske et al., 2017) and depression (Fakhoury, 2016). One of the less studied roles of 5‐HT is how the neurotransmitter serves as a modulator of attention, arousal, and motivation (Gu, 2007). Studies have shown that visual cortex excitability is modulated by electrical stimulation of the origin of serotonergic projections, the raphe nuclei (Gasanov, Mamedov, & Samedova, 1989) and by direct application of 5‐HT (Waterhouse, Ausim Azizi, Burne, & Woodward, 1990). Also, administration of the 5‐HT reuptake inhibitor fluoxetine facilitates short‐term plasticity in the primary visual cortex of cats (Bachatene, Bharmauria, Cattan, & Molotchnikoff, 2013) and increases threshold of conscious visual perception in humans (Lansner et al., 2019), supporting the involvement of 5‐HT in visual attention. Furthermore, human neuroimaging studies with acute selective serotonin reuptake inhibitor administration suggest that 5‐HT is involved in response regulation or integration of external stimuli (I. M. Anderson et al., 2007; Bigos et al., 2008; Del‐Ben et al., 2005).

External stimulation was used in a seminal proof‐of‐concept neuroimaging study, where Belliveau et al. showed that magnetic resonance imaging (MRI) can measure neuronal activation induced by a strong visual stimulus (Belliveau et al., 1991). Since then, visual stimulation with checkerboard has become known as one of the most potent activation paradigms in functional MRI (fMRI) studies. Less potent visual stimuli, such as the face‐paradigms with different emotional expressions, were used early on to identify regions of the brains with increased cerebral blood flow (CBF) (Gur, Skolnick, & Gur, 1994) and later to create neurofunctional maps of emotion using blood oxygenated level‐dependent fMRI (Fusar‐Poli et al., 2009). Engaging and relatable visual stimuli motivated participant attention and have also been used to study the neural response to seeing loved partners (Zeki & Romaya, 2010).

Since the development of fMRI in the early 90s, the technology has rapidly advanced and with hybrid positron emission tomography (PET)/MRI it is now possible to simultaneous assess brain neurochemistry, activity, and functional characterization of drugs in the living brain (Sander, Hansen, & Wey, 2020). Another unique feature is its ability to investigate the temporal correlation between neurochemistry and neuronal activity, and to investigate the brain networks and/or connectivity during a physiological or cognitive task. A prerequisite for measuring changes in neurochemistry is a PET radiotracer sensitive to changes in neurotransmitter concentration. The selective 5‐HT1B receptor radiotracer [^11^C]AZ10419369, represents such a tracer: Pharmacological experiments in pigs (Jørgensen et al., 2018), nonhuman primates (Yang, Takano, Halldin, Farde, & Finnema, 2018), and humans (Nord, Finnema, Halldin, & Farde, 2013) have confirmed that this tracer is sensitive to the release of endogenous 5‐HT in the brain. In a recent combined PET and microdialysis study in pigs it was demonstrated that the sensitivity of [^11^C]AZ10419369 PET for detecting changes in interstitial 5‐HT is in the same order of magnitude as [^11^C]raclopride for measuring changes in dopamine levels in vivo (Jørgensen et al., 2018).

The aim of this study was to investigate the change in 5‐HT following a visual stimulus and the associated neural responses. This study represents one of the first PET/MR imaging studies investigating neurotransmitter release using a nonpharmacological stimulus. We hypothesized that in addition to an increased CBF in thalamus and visual cortex, presentation of participant‐specific salient images would release 5‐HT, which we would measure as a decrease in [^11^C]AZ10419369 binding.

## 2 MATERIALS AND METHODS

### 2.1 Participants

Eleven healthy volunteers were recruited from a database of healthy individuals. All participants were selected according to the following criteria: (a) absence of current or lifetime history of major psychiatric disorders (major depressive disorder, bipolar disorder, or psychotic symptomatology); (b) absence of symptomatic medical or neurological illness, head trauma with loss of consciousness for more than 30 min, severe visual or hearing impairment, and contraindications for MRI; (c) absence of current substance or alcohol abuse; and (d) no pregnancy or nursing. All included participants had normal blood biochemistry were tested negative on a urine drug screen (Rapid Response Multi‐Drug; BTNX Inc., Toronto, ON, Canada) on the day of scanning and had an unremarkable MRI of the brain. The study was approved by the local ethical committee (Copenhagen, Denmark; reference H‐2‐2014‐070 and the Danish Medicines Agency [EudraCT‐nr.: 2015‐002861‐52]). All participants provided written informed consent following full description of the procedures and received a monetary compensation for their participation.

### 2.2 Study design

**FIGURE 1:**
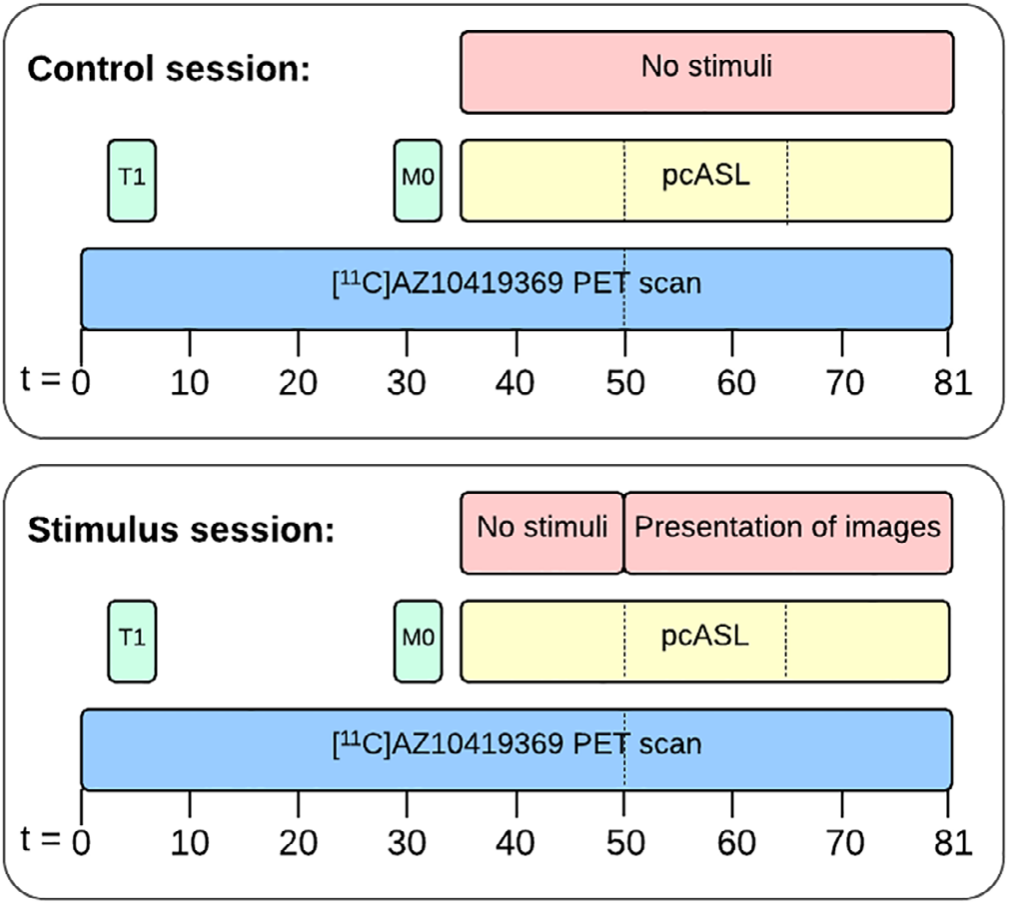
Schematic overview of the experimental design of the two PET/MR imaging sessions. In the stimuli session, presentation of their autobiographical images started after 50 min and lasted to the end of the scan. In the control session, participants had their eyes closed. For PET, two nondisplaceable binding potentials were calculated. BP0: *t* = 0–50 min and BP1: *t* = 50–81 min. For the pcASL data, CBF was calculated in three blocks of 15 min: CBF_35–50 min_, CBF_50–65 min_ and CBF_65–80 min_. Dashed lines indicate the time included in each analysis. BP, binding potential; CBF, cerebral blood flow; pcASL, pseudo‐continuous arterial spin labeling; PET/MR, positron emission tomography/magnetic resonance

Seven participants were scanned twice in alternating sequence, either with their eyes closed (control session) or during a visual stimulus (stimulus session) (Figure 1). The control session was conducted to validate the use of the extended simplified reference tissue model, which was used for quantification (see also below). Four additional participants were scanned with the stimulus paradigm only. To ensure optimal engagement and attention, participants selected among their own photos; they were told to choose personal and emotionally positive pictures (e.g., pictures with family, loved ones, on holiday, etc.). Images were presented for 30 s with a 5‐s interval between pictures (i.e., 52 pictures shown). The order of participant‐selected images was randomized and presented using E‐prime software (Psychology Software Tools, Sharpsburg, PA). In the control session, they were instructed to keep their eyes closed and to avoid falling asleep. After the PET/MR scan, participants completed a simple questionnaire asking whether the participant had slept during the scan. Participants in the control session scored on average 0.85 (range 0–3, *n* = 7) and participants in the stimulus session scored on average 0.6 (range 0–2, *n* = 10) on a scale from 0 to 4, where 0 corresponded to no sleep and 4 corresponded to high degree of sleeping during the scan. No outliers were identified in the two groups using the Grubbs test (*α* = .05). In the stimulus session, participants were instructed to focus on the images and dwell on the positive memories associated with each presented image. We could not evaluate whether the subjects had their eyes closed or open during the first part of the stimulus part (35–50 min). The scanner environment was very dark before the initiation of the stimulus resulting in limited visual input and brain activity in the occipital cortex. Thereby, a strong contrast in visual input was achieved before and after the initiation of the visual paradigm.

### 2.3 Imaging

Participants underwent a simultaneous PET/MR scan in a Siemens Biograph mMR scanner. MR imaging consisted of a T1 MPRAGE, M0 and pseudo‐continuous arterial spin labeling (pcASL). PET imaging was initiated simultaneously with the intravenous bolus injection of the 5‐HT_1B_ receptor radioligand [^11^C]AZ10419369. The pcASL sequence was started 35 min after the injection of the radioligand. After 15 min of baseline measurement, the presentation of images started, resulting in 30‐min pcASL data being acquired during the presentation of images (see Figure 1).

### 2.4 PET image reconstruction

5‐HT_1B_ receptor binding was measured with the radioligand [^11^C]AZ10419369, which was synthesized as described previously (da Cunha‐Bang et al., 2017). An intravenous bolus injection of the radioligand was given over 20 s, followed by 81‐min dynamic data acquisition. Data were arranged into 43 frames (12 × 10, 6 × 20, 6 × 60, 8 × 120, and 11 × 300 s) and reconstructed using the ordered subset expectation maximization method with a coregistered computed tomography (CT)‐based attenuation correction map, 4 iterations, 21 subsets, and 4 mm FWHM filter. For CT‐based attenuation correction, a low‐dose CT image (120 kVp, 36–40 mAs, less than 0.5 mSv effective dose) of the head was performed on Siemens Biograph TruePoint 40/64 or mCT 64 PET/CT scanners based on availability, with bilinear scaling to 511 keV (Carney et al., 2006), with the minor adaptation of the method as implemented on Siemens Biograph PET/CT (Ladefoged et al., 2015).

### 2.5 Preprocessing of PET data

All PET images were motion corrected using the Automated Image Registration (v. 5.2.5, LONI, UCLA) software where all frames were aligned to the first 5‐min frame (28.5‐min postradioligand injection). PET images were coregistered and aligned to the subject's T1‐weighted MRI image. Regions of interests (ROIs) were automatically delineated using PVElab (Svarer et al., 2005) (Neurobiology Research Unit [NRU], Copenhagen, Denmark; https://nru.dk/pveout) and SPM8 (Wellcome Trust Centre for Neuroimaging, London, UK; http://www.fil.ion.ucl.ac.uk/spm).

### 2.6 Magnetic resonance imaging

MRI scans were acquired simultaneously with the PET scans (see Figure 1) using a 12‐channel receive head and neck coil provided by the vendor. A high‐resolution structural 3D T1 MPRAGE scan covering the entire head using 192 sagittal slices was acquired for anatomical reference and normalization (echo time [TE]: 2.44 ms; repetition time [TR]: 1900 ms; flip Angle: 9°; inversion time [TI]: 900 ms; field of view [FOV]: 512 × 512 × 192 with an interpolated resolution of 0.5 × 0.5 × 1.0 mm^3^). ASL was acquired using a pseudo‐continuous labeling for 1730 ms and a postlabeling delay of 1,500 ms followed by a 2D Echo Planar Imaging readout of 20 axial slices (TE: 12 ms; TR: 4000 ms; flip angle: 90°; pairs: 338; slice readout duration time: 35 ms) (Kilroy et al., 2014). For calibration of the ASL signal, an M0 scan was acquired using the same imaging parameters as for the ASL sequence with a repetition time of 20 s.

### 2.7 Data analysis

#### 2.7.1 Descriptive statistics

Subject differences between scan sessions (weight and age) and parameters related to the radioligand (injected dose in MBq and MBq/kg, injected mass in μg and μg/kg) were tested using the Wilcoxon matched‐pairs signed rank test. The Wilcoxon signed‐ranked test was chosen because of the limited number of subjects in our sample and in the presence of not‐normally distributed variables the *t* test may not provide an appropriate control of the Type 1 error. As mentioned in the literature (Fagerland, 2012), Wilcoxon tests are not only sensitive to difference in mean or median but may also detect difference in spread.

#### 2.7.2 Analysis of pcASL data

All analyses of the pcASL data were carried out using FSL 5.0.11 (FMRIB, Oxford, UK). The total 45‐min pcASL data were broken into three frames, each of 15 min length. A set of shorter 5‐min frames of CBF was also attempted; however, this resulted in too large variation in CBF values. Pairwise subtraction, motion correction, standard space normalization, and kinetic modeling were performed using BASIL with default values for T1, T1b, and bolus arrival time. Slice timing was corrected using a slice readout duration of 35 ms. Statistical comparison of the three blocks was performed by comparing the mean value at each voxel between each 15‐min time frame within a session using permutation testing (FSL, RANDOMIZE; Winkler, Ridgway, Webster, Smith, & Nichols, 2014) utilizing only within participant permutations and controlling for multiple comparisons using family‐wise error rate.

In addition to the voxel‐based analysis, we also performed an ROI‐based analysis. To apply the same regions for PET and MR analyses, the subject‐specific regions from the NRU atlas were transformed into T1 space, using the transformation matrix between the PET and T1 images. This allowed for extraction of CBF values in regions identical to the regions used for quantification of receptor binding. The CBF values were analyzed using a separate linear mixed‐effect model for each region, modeling how session type and time relate to CBF using six regression parameters (one for each coupled session type, time). Five random effects were used to model the variance and covariance of the CBF measurements within an individual. The variance–covariance of the random effect was assumed to be block diagonal, with one block for random effects relative to time and another for the random effects relative to region. Within a block, no assumption was made (unstructured variance–covariance matrix). To test whether CBF was changed after starting the presentation of images, we assessed the change in CBF over time in the stimulus session (CBF_35–50 min_ vs. CBF_50–65 min_ and CBF_35–50 min_ vs. CBF_65–80 min_, within‐session analysis) and compared the change in CBF between the control and the stimulus session (e.g., CBFstimulus_50–65 min_ – CBFstimulus_35–50 min_ vs. CBFcontrol_50–65 min_ – CBFcontrol_35–50min_, between‐session analysis) using Wald tests. To control for Type 1 errors in this sample with a small number of observations, we modified the standard asymptotic results on the Wald statistic using the method proposed by Kenward and Roger (1997). No covariates were included in the models analyzing pcASL data. The Bonferroni–Holm method was used to correct for multiple comparisons; *p* < .05 was considered statistically significant.

#### 2.7.3 Analysis of PET data

PET data were quantified using the extended simplified reference tissue model (ESRTM) (Zhou et al., 2006) using the cerebellum as a reference region. ESTRM returns two nondisplaceable binding potential (BP_ND_) values: one for the initial (unstimulated) phase of the scan (0–50 min, BP0) and one for the intervention phase (50–81 min, BP1). It was not possible to investigate the changes in BPs in the thalamus because the modeling of the data did not fulfill our quality requirements (<15% coefficient of variation on BP0 and BP1 estimates). To account for the bias between BP0 and BP1 (Hansen, Da Cunha‐Bang, Svarer, & Knudsen, 2014), we normalized the BP_ND_ values to the BP in the sensory motor cortex according to Equation (1). We calculated the percent difference between the two normalized BPs as:

(1) $$\mathrm{∆BP}=\frac{\frac{\mathrm{BP}1_{\mathrm{ROI}}}{\mathrm{BP}1_{\text{sensory motor cortex}}}-\frac{\mathrm{BP}0_{\mathrm{ROI}}}{\mathrm{BP}0_{\text{sensory motor cortex}}}}{\frac{\mathrm{BP}0_{\mathrm{ROI}}}{\mathrm{BP}0_{\text{sensory motor cortex}}}}\times 100.$$

For the within‐session analysis, we did not have missing values and therefore we used one‐sided paired *t* test for analysis of differences in BP_ND_ values. Similar to the ROI‐based pcASL data, differences in BP_ND_ values across sessions were analyzed using a linear mixed‐effect model. No covariates were included in the analysis. *p* Values for both within‐session analysis (three ROIs, two sessions = six tests) and between‐session analysis (three ROIs = three tests) were corrected for multiple comparisons using the Bonferroni–Holm method; *p* < 0.05 was considered statistically significant.

#### 2.7.4 Association between changes in CBF and 5‐HT_1B_ receptor binding

To investigate the association between the changes in CBF and 5‐HT_1B_ receptor binding, a linear mixed‐effect model was used. The random effect was used to model the correlation across sessions and a different variance was considered at each session (unstructured covariance matrix). The regression was forced through origin (0,0) based upon the hypothesis, that with no change in 5‐HT release, no changes in CBF would be observed.

Statistical analyses were performed using the R software (R Code Team, 2018). Graphs were created in GraphPad Prism 7.01 (GraphPad Software, La Jolla, CA).

## 3 RESULTS

A summary of participant characteristics is provided in Table S1.

### 3.1 Analysis of pcASL data

#### 3.1.1 Within‐session analysis

**FIGURE 2:**
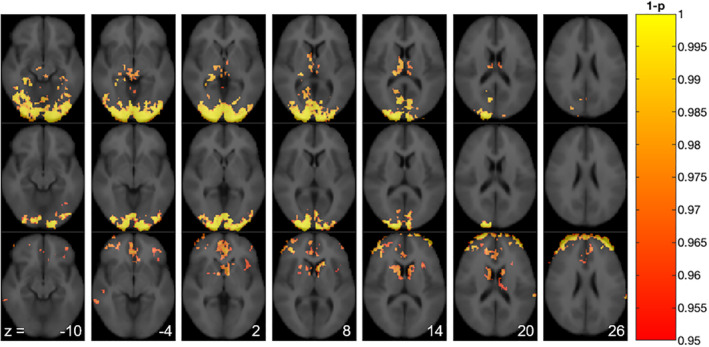
Significance maps showing voxels with changes in CBF in the stimulus session (upper row: 50–65 min > 35–50 min; middle row: 65–80 min > 35–50 min; lower row: 50–65 min > 65–80 min, *n* = 11). Numbers indicate *z*‐axis in mm. CBF, cerebral blood flow

When contrasting the two stimuli frames (CBF_50–65 min_ and CBF_65–80 min_) to the preceding baseline frame (CBF_35–50 min_), voxel‐based analysis of the pcASL data in the stimulus session identified clusters with significantly increased CBF in the occipital cortex and thalamus (Figure 2, top and middle row). When contrasting CBF_50–65 min_ to CBF_65–80 min_, we found that CBF decreased in the caudate nucleus and anterior cingulate cortex in the late part of the stimulus session (Figure 2, lower row). In the control session, no significant differences in CBF were found when contrasting the baseline frame (CBF_35–50 min_) to the two later frames (CBF_50–65 min_ and CBF_65–80 min_). Similarly, no significant changes in CBF were found between the two latter frames.

In an ROI‐based and within‐session analysis of CBF values (Table 1), we found that CBF was significantly increased in the occipital cortex in the stimulus session with a mean change ± *SD* of +6.4 ± 1.3 mL/100 g/min, *p* = .001 for CBF_50–65 min_ and +5.4 ± 1.5, *p* = .015 for CBF_65–80 min_ compared to the preceding baseline frame (CBF_35–50 min_). CBF was also increased in the thalamus in the early part of the stimulus session (CBF_50–65 min_: +4.9 ± 1.4 mL/100 g/min, *p* = .015) compared to the baseline frame. In the anterior cingulate cortex, we found that CBF decreased in the latter part of the stimulus session (CBF_65–80 min_: −3.2 ± 1.1 mL/100 g/min, *p* = .03) compared to the preceding baseline frame. In contrast to the voxel‐based analysis, no significant changes in CBF were seen in the caudate nucleus. For individual regional CBF values, see Figures S1 and S2.

**TABLE 1:** Cerebral blood flow (CBF, average ± *SE*) values from the stimulus session at different time points and the *P* values after a linear mixed‐model analysis comparing CBF_50–65 min_ or CBF_65–80 min_ to the baseline measurements, CBF_35–50 min_ (within‐session analysis)

Region of interest	Time (min)	Baseline CBF (ml 100 g^−1^ min^−1^)	Stimulus CBF (ml 100 g^−1^ min^−1^)	Difference (ml 100 g^−1^ min^−1^)	*SE*	*P* Value	Adjusted *P* value
Occipital cortex	35–50	48.7 ± 8.07					
	50–65		55.1 ± 10.8	6.4	1.30	<.001	.001
	65–80		54.1 ± 11.0	5.4	1.45	.002	.015
Thalamus	35–50	49.4 ± 10.7					
	50–65		54.3 ± 8.45	4.93	1.41	.002	.015
	65–80		52.7 ± 10.4	3.33	1.51	.040	.119
Caudate	35–50	56.8 ± 14.3					
	50–65		58.3 ± 12.6	1.58	1.00	.128	.256
	65–80		54.0 ± 13.0	−2.80	1.15	.026	.104
Anterior cingulate cortex	35–50	62.5 ± 14.9					
	50–65		63.8 ± 13.12	1.31	0.94	.175	.256
	65–80		59.3 ± 12.25	−3.22	1.06	.008	.040

#### 3.1.2 Between‐session analysis

As a sensitivity analysis, we also performed a between‐session analysis in which we examined the changes in the stimulus session versus the changes during the control session. This analysis corroborated our first analysis: while generally smaller (in absolute value), the differences had the same signs as in the main analysis. *SE* values were also generally larger and statistical significance was only reached when testing the change in CBF in the occipital cortex (see Table S2).

### 3.2 Analysis of PET data

A representative example of [^11^C]AZ10419369 time–activity curve from a stimulus session is shown in Figure S3.

#### 3.2.1 Within‐session analysis

We found no significant changes between normalized BP0 and BP1 in the control session. In the stimulus session we found significant changes between the normalized BP0 and BP1 in the occipital cortex (*p* = .02) and anterior cingulate cortex (*p* = .02).

#### 3.2.2 Between‐session analysis

**FIGURE 3:**
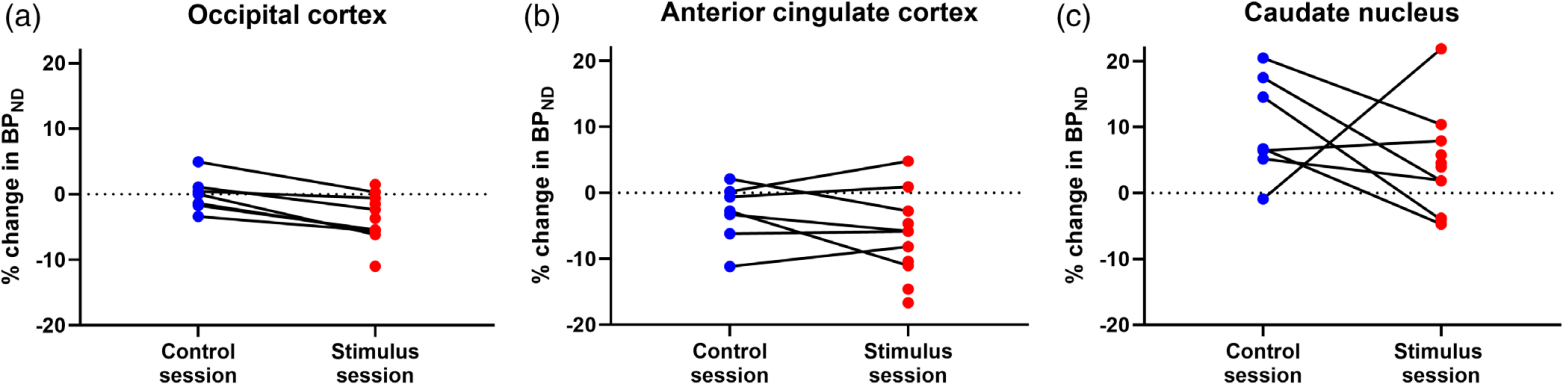
Regional % differences in normalized nondisplaceable binding potential (BP_ND_) in the occipital cortex (a), anterior cingulate cortex (b) and caudate nucleus (c). “Stimulus session” represents the scan in which participants had image presentation. “Control session” represents the scan in which participants had no stimulation

The change between normalized BP0 and BP1 in the occipital cortex differed significantly (*p* = .004) between the two sessions: the change from BP0 to BP1 in the visual stimulus session was −3.6 ± 3.6% (Figure 3a), whereas there was little change from BP0 to BP1 in the control session (−0.0 ± 2.7%). All values represent mean percent change in BPs ± *SE*. No session differences were found in the anterior cingulate cortex (*p* = .30, Figure 3b) or in the caudate nucleus (*p* = .30, Figure 3c). No significant difference was found between normalized BP0 (control session) and normalized BP0 (stimulus session) in the occipital cortex (*p* = .59, *n* = 7, paired *t* test).

### 3.3 Association between changes in CBF and 5‐HT_1B_ receptor binding

**FIGURE 4:**
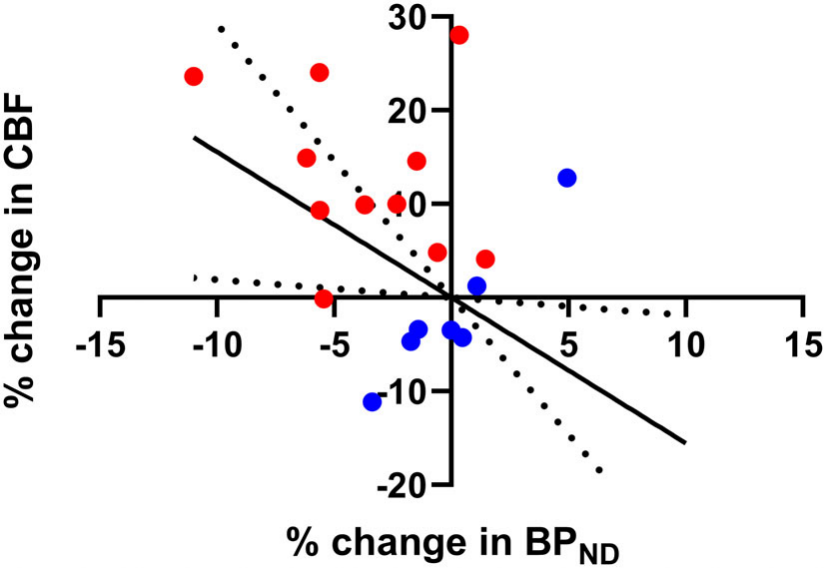
Linear correlation between the change in normalized non‐displaceable binding potential (BP_ND_) and the change in cerebral blood flow (CBF) in the occipital cortex: CBF_35–50 min_ versus CBF_50–65 min_. Blue symbols represent the control session and red symbols represent the stimulus session. Dotted lines represent the lower and upper bound of 95% confidence intervals of the % change in CBF for various % change in BP_ND_

Using a linear mixed‐effect model including all data points (control and stimulus session), an association (*p* = .004) was found between the percent change in normalized BP_ND_ and the percent change in CBF in the occipital cortex (Figure 4) with a regression coefficient of −2.48 ± 0.59 (mean ± *SE*). The regression remained significant (*p* = .036) when not forcing the regression through the origin (0,0). Also, without the control session data points, the association was statistically significant (*p* = .01) and it had a similar regression coefficient of −2.20 ± 0.70 (mean ± *SE*).

## 4 DISCUSSION

In response to the presentation of positive autobiographical images, we found as expected an increased CBF in the occipital cortex; we also found a decrease in BP_ND_ in the occipital cortex. Our interpretation is that the visual stimulation causes synaptic 5‐HT to increase, which causes a displacement of the radioligand. This is conceivable given studies have shown that [^11^C]AZ10419369 is sensitive to pharmacologically induced changes in 5‐HT in animals (Jørgensen et al., 2018) and humans (Nord et al., 2013). To our knowledge, this is the first study demonstrating cerebral 5‐HT release in response to a nonpharmacological intervention.

The use of hybrid PET/MRI systems decrease intrasubject variability by obviating separate measurements with PET and MR. In addition, with an experimental design like ours that involves a within‐scan intervention, this variability is further reduced. Accordingly, factors such as attentional bias, circadian rhythm and state measures do not have to be considered when analyzing correlations between the PET and MR results. The current study served as proof‐of‐principle and we therefore chose to perform scans with and without stimulus to validate the study design. The results show that two scans are not necessary for future scans with [^11^C]AZ10419369. Because of a bias in the global brain signal between the initial versus the last part of the PET scan, we chose to normalize the BP from our regions of interest to that of the sensory‐motor cortex. Normalization of PET macroparameters was used in an experimentally similar PET study, in which flow (measured with [^15^O]water) and kinetic parameters of [^11^C]flumazenil was studied with and without visual stimuli (Holthoff, Koeppe, Frey, Paradise, & Kuhl, 1991).

In this study, we find a statistically significant correlation between the changes in CBF and changes in BP_ND_ when participants are presented with autobiographical images. This linear correlation should be interpreted cautiously and does not necessarily imply causation. However, we speculate a mechanism of action in which the release of 5‐HT and subsequent activation of receptors leads to a downstream activation of the neurons and a subsequent increase in energy demand. Below, we present evidence that supports this interpretation.

A number of the 5‐HT receptors are highly expressed by the majority of excitatory neurons in the occipital cortex (Beliveau et al., 2017; Watakabe et al., 2009). Thus, the 5‐HT release will result in a net effect between inhibition and activation mediated by the different receptor types. An electrophysiological study showed that a global activation of 5‐HT receptors in the visual cortex by endogenous 5‐HT shifts the excitation‐inhibition balance in favor of excitation (William Moreau, Amar, Le Roux, Morel, & Fossier, 2010), which may explain the increased energy demand and consequently increased blood flow. Long‐term use of serotonin transporter inhibitor 3,4‐methylene‐dioxymethamphetamine (also known as “Ecstasy”) results in altered visual perception thought to reflect serotonergic dysfunction in the occipital lobe (White, Brown, & Edwards, 2013). Contrary to this, in vitro experiments shows that stimulation of the 5‐HT_1B_ receptors with both 5‐HT and selective agonists inhibited excitatory synaptic transmission in rat cingulate cortex (Tanaka & North, 1993). Multiple electrophysiological studies demonstrate that synaptically released 5‐HT induces presynaptic inhibition of glutamate transmission by acting on 5‐HT_1B_ receptors (Lemos et al., 2006; Lu, Lin, Huang, & Wang, 2018; Mlinar, Falsini, & Corradetti, 2003). Coactivation of 5‐HT_1A_ and 5‐HT_7_ receptors also induces attenuation of glutamatergic synaptic transmission in the rat visual cortex (Li et al., 2018). In summary, given the many different 5‐HT receptors and their complex G‐protein coupling, we cannot identity which of the receptors are specifically involved in the mechanism of action here. Although the precise balance of 5‐HT receptors mediating the 5‐HT release effects is unknown, the findings here provide novel evidence that visual stimulation induces a release of 5‐HT that is correlated with blood flow.

The role for 5‐HT in the response to emotional stimuli is based primarily on the neural responses to pharmacologically increased serotonin levels (reviewed by Cools, Roberts, & Robbins, 2008; Wessa & Lois, 2015). Acute tryptophan depletion, associated with low brain 5‐HT levels, results in an increased neural response to emotional and in particular sad words as measured with fMRI (Roiser et al., 2008). Contrary to this, decreased catecholamine transmission results in decreased CBF in the posterior cingulate cortex in response to fearful faces as measured with PET (Homan, Drevets, & Hasler, 2014). Of relevance to the current study, the neural response to a stimulus also depends on the valence and the reward associated with it: Stimuli associated with high reward produce stronger visually evoked responses compared to previously unrewarded or less valuable stimuli (B. A. Anderson, 2019). Given that the stimuli used in the current study consist of autobiographical images with strong positive valence and high reward, we hypothesized to find 5‐HT release in areas involved in emotional processing, for example, ventral striatum and anterior cingulate cortex (Etkin, Egner, & Kalisch, 2011). However, we were not able to detect statistically significant changes in 5‐HT release. Nevertheless, we did find changes in CBF in these regions, and this finding is corroborated by a PET study measuring CBF in response to schematic (“hot”) or propositional (“cold”) emotional information. Here, the brain areas that are more active in the “hot” compared to the “cold” processing mode corresponded to the prefrontal and dorsal anterior cingulate cortices (Schaefer et al., 2003). We believe that these changes in CBF could be specific to the emotional salience of the stimuli, as no activation of anterior cingulate nor the ventral striatum was found during a powerful checkerboard stimuli (Hougaard et al., 2015).

In the voxel‐wise analysis of the CBF response, we observed decreased CBF in the caudate nucleus in the late part of the stimulus session (CBF_50–65 min_ > CBF_65–80 min_). This could reflect a reward‐induced dopamine release, whereby dopamine stimulates primarily the D_2_ and D_3_ receptors and causes an inhibitory neural response (Sander, Hooker, Catana, Rosen, & Mandeville, 2016). In disfavor of this interpretation is the fact that the response is delayed compared to the onset of image presentation. It is also possible that the responses seen in the caudate nucleus (and anterior cingulate cortex) relate to visual attention. Two recent PET studies, where dopamine release was measured while participants performed visual search tasks, suggest a relationship between dopamine signaling within the striatum and the control of visual attention (B. A. Anderson et al., 2016, 2017).

The change in radiotracer binding due to neurotransmitter release is defined as $\text{occupancy}=\frac{{∆F}_{\mathrm{NT}}}{K_{\mathrm{NT}}+F_{\mathrm{NT}}+{∆F}_{\mathrm{NT}}}$, where *F*_NT_ is free neurotransmitter, Δ*F*_NT_ is the change in neurotransmitter, and *K*_NT_ is the affinity of the neurotransmitter for the receptor. Thus, measuring signal change in response to a physiological or pharmacological challenge is independent of radiotracer characteristics and expression level of the receptor (*B*_max_), and solely depends on the affinity of the endogenous neurotransmitter for the target receptor. Pharmacological interventions such as escitalopram and fenfluramine cause large increases in synaptic 5‐HT concentration which results in decreased binding of [^11^C]AZ10419369. In nonhuman primates, the measured occupancy was found to be to be 12 ± 8 and 39 ± 8% for escitalopram (Nord et al., 2013) and fenfluramine (Finnema et al., JCBFM, 2012), respectively. These occupancies are measured in high‐binding regions but identification of a robust signal change in low‐binding regions can be more challenging. In comparison, a visual paradigm is assumed to be a milder stimulus causing a smaller release of 5‐HT and it will therefore also result in lower occupancies. The 4.2% occupancy measured in the current study therefore seems comparable to previous studies with [^11^C]AZ10419369.

Delivery of radioligand to the brain tissue depends partly on the flow of blood through the capillaries. Although we find increased blood flow in some brain regions, we find it unlikely that a change in CBF is influencing the radioligand binding because of the low extraction of [^11^C]AZ10419369. Sander et al. demonstrated with both in vivo experiments and data simulations that radioligands with low K_1_ values are not very sensitive to changes in CBF (Sander et al., 2019). The K_1_ value of [^11^C]AZ10419369 is in the range of 0.05–0.13 mL/cm^3^ (Varnas et al., 2011). That is, even a 100% increase in CBF is expected to result in less than 1% change in BP (Sander et al., 2019). Holthoff et al. found that visual stimuli increased CBF by 22% in the occipital cortex and consequently increased K_1_ of [^11^C]flumazenil by 21% in the same region. However, no significant change in distribution volumes was found (Holthoff et al., 1991). We found a maximal regional change in CBF of 15%, making it very unlikely that the CBF change affected the BP.

The current study is not without limitations. Although we included a small number of participants, it has the advantage of paired design. We chose to analyze the data using a linear mixed‐effect model because this is a more powerful approach that takes missing data points into consideration. As also mentioned in the method section, we controlled for type 1 errors by modifying the standard asymptotic results on the Wald statistic using the method proposed by Kenward and Roger (1997). In summary, we are confident in the results despite the small sample size.

Another limitation to this study is that we did not perfectly control for participants having their eyes open during the entire scan. This resulted in the participants in the control session likely having their eyes closed whereas eyes were kept open in the stimulus session. We account for this discrepancy by performing the between‐session analysis of CBF on relative instead of absolute values.

Cerebellum is a validated reference region for quantification of 5‐HT_1B_ receptor availability using [^11^C]AZ10419369 (Ganz et al., 2017). In the quantification of PET data from the control session, we found that BP1 < BP0 in cortical regions which we believe are driven by imperfections in the kinetic model (Zhou et al., 2006). To account for this bias, we normalized the regional BP0 and BP1 to the corresponding BP0 or BP1 in the sensory motor cortex. The sensory motor cortex is a region that was not activated while the participants are inside the scanner. We did not find any significant changes in CBF in the sensory motor cortex (control session: *p* = .55, Figure S2e; stimulus session: *p* = .46, Figure S1e; paired *t* tests) indicating that this region was indeed not activated during the scan. In this study, we found that the [^11^C]AZ10419369 tracer kinetics are slower in subcortical regions, such as the caudate. Although the model fits from this ROI were all within our quality criteria (maximum 15% coefficient of variation on BP0 and BP1) the outcome measures were more variable then those seen in cortical regions.

We encourage development of tracers that are sensitive for changes in neurotransmitters but importantly also the development of quantification models that works well for a variety of tracers and interventions. Many of the current models are developed and validated specifically for [^11^C]raclopride, but they may not apply equally well to other radioligands.

## 5 CONCLUSIONS

In this experimental setup with autobiographical images as visual input, we found small but significant changes in 5‐HT_1B_ receptor binding and CBF in the occipital cortex. We speculate that using more powerful physiological stimuli would cause larger 5‐HT release and consequently increase the changes in binding and CBF. The association between the changes in receptor binding and CBF suggest a mechanism in which 5‐HT is involved in either attention or processing of visual material. To the best of our knowledge, this is the first time that simultaneous changes in CBF and 5‐HT levels in response to physiological stimuli have been measured. Together, these findings provide a valuable, methodological proof‐of‐concept and further inform how 5‐HT neurotransmission shapes visual processing and further studies are warranted to investigate the role of 5‐HT in processing of other sensory stimuli.

## CONFLICT OF INTEREST

The authors declare no potential conflict of interest.

## Supporting information


**APPENDIX** **S1.** Supporting Information
**TABLE S1**: Subject characteristics and PET variables. Wilcoxon matched‐pairs signed rank test. No statistical differences between scan sessions were found in parameters related to the radioligand
**FIGURE S1**. Line‐plot showing the cerebral blood flow (CBF) in occipital cortex (a), thalamus (b), caudate nucleus (c), anterior cingulate cortex (d), and sensory motor cortex (e) in subjects undergoing the PET/MR stimulus session. The visual stimuli were initiated at *t* = 50 min. *p < .05; **p < 0.01; ***p > 0.001.
**FIGURE S2.** Line‐plot showing the cerebral blood flow (CBF) in occipital cortex (a), thalamus (b), caudate nucleus (c), anterior cingulate cortex (d), and sensory motor cortex (e) in subjects undergoing the PET/MR control session.
**TABLE S2:** Summary of the changes in CBF in the control session versus the changes in CBF in stimuli session
**FIGURE S3.** A representative [^11^C]AZ10419369 time‐activity curve from a stimulus session for five different regions of interest. Symbols represent measured radioactivity normalized to injected dose and weight of the participant yielding standardized uptake value (SUV). Lines represent model fit of the extended simplified reference tissue model. The vertical dotted line represents the onset of the visual stimuli.

## Data Availability

The data that support the findings of this study are available on request from the corresponding author. The data are not publicly available due to privacy or ethical restrictions.
