# Supplementary material for: Visual stimuli induce serotonin release in occipital cortex: A simultaneous positron emission tomography/magnetic resonance imaging study
Source: Hum Brain Mapp. 2020 Aug 19;41(16):4753–63. doi: 10.1002/hbm.25156 (PMC7555083; doi:10.1002/hbm.25156)
Supplement: Supplementary file 1 — APPENDIX S1. Supporting Information TABLE S1: Subject characteristics and PET variables. Wilcoxon matched‐pairs signed rank test. No statistical differences between scan sessions were found in parameters related to the radioligand FIGURE S1. Line‐plot showing the cerebral blood flow (CBF) in occipital cortex (a), thalamus (b), caudate nucleus (c), anterior cingulate cortex (d), and sensory motor cortex (e) in subjects undergoing the PET/MR stimulus session. The visual stimuli were initiated at t = 50 min. *p < .05; **p < 0.01; ***p > 0.001. FIGURE S2. Line‐plot showing the cerebral blood flow (CBF) in occipital cortex (a), thalamus (b), caudate nucleus (c), anterior cingulate cortex (d), and sensory motor cortex (e) in subjects undergoing the PET/MR control session. TABLE S2: Summary of the changes in CBF in the control session versus the changes in CBF in stimuli session FIGURE S3. A representative [11C]AZ10419369 time‐activity curve from a stimulus session for five different regions of interest. Symbols represent measured radioactivity normalized to injected dose and weight of the participant yielding standardized uptake value (SUV). Lines represent model fit of the extended simplified reference tissue model. The vertical dotted line represents the onset of the visual stimuli. [file HBM-41-4753-s001.docx]

**Supplementary information**

**Visual stimuli induce serotonin release in occipital cortex: A simultaneous PET/MRI study**

Hanne Demant Hansen^1^, Ulrich Lindberg^2^, Brice Ozenne^1,3^, Patrick MacDonald Fisher^1^, Annette Johansen^1,4^, Claus Svarer^1^, Sune Høgild Keller^2^, Adam Espe Hansen^2^, Gitte Moos Knudsen^1,4^

- 1. *Neurobiology Research Unit and NeuroPharm, Copenhagen University Hospital, Rigshospitalet, Copenhagen, Denmark*
  2. *Department of Clinical Physiology, Nuclear Medicine and PET, Copenhagen University Hospital, Rigshospitalet, Copenhagen, Denmark*
  3. *Department of Public Health, Section of Biostatistics, University of Copenhagen, 5 Øster Farimagsgade, 1014 Copenhagen K, Denmark*
  4. *Faculty of Health and Medical Sciences. University of Copenhagen. 3 Blegdamsvej. 2100 Copenhagen, Denmark*

**Supplementary table 1:** Subject characteristics and PET variables. Wilcoxon matched-pairs signed rank test. No statistical differences between scan sessions were found in parameters related to the radioligand.

|  | Control | Intervention | P value |
| --- | --- | --- | --- |
| Number of subjects  (men/female) | 7  (1/6) | 11  (1/10) |  |
| Age | 27.2 ± 4.5 | 25.4 ± 4.4 | 0.77 |
| Weight (kg) | 71.0 ± 12.2 | 68.5 ± 11.0 | 0.13 |
| Injected dose (MBq) | 589 ± 58 | 544 ± 86 | 0.30 |
| Injected dose (MBq/kg) | 8.56 ± 1.87 | 7.70 ± 2.31 | 0.69 |
| Molar radioactivity (GBq/μmol) | 139 ± 67 | 153 ± 88 | 0.58 |
| [^11^C]AZ injected mass (μg) | 2.54 ± 1.47 | 2.55 ± 1.88 | 0.69 |
| [^11^C]AZ injected mass (ug/kg) | 0.03 ± 0.02 | 0.04 ± 0.02 | 0.58 |

**Supplementary figure 1**. Line-plot showing the cerebral blood flow (CBF) in occipital cortex (A), thalamus (B), caudate nucleus (C), anterior cingulate cortex (D) and sensory motor cortex (E) in subjects undergoing the PET/MR stimulus session. The visual stimuli were initiated at t=50 min. * represent p<0.05; ** represent p<0.01; *** represent p>0.001.

**Supplementary figure 2.** Line-plot showing the cerebral blood flow (CBF) in occipital cortex (A), thalamus (B), caudate nucleus (C), anterior cingulate cortex (D) and sensory motor cortex (E) in subjects undergoing the PET/MR control session.

**Supplementary table 2:** Summary of the changes in CBF in the control session versus the changes in CBF in stimuli session.

| Region | Time (min) | Change in CBF control | Change in CBF stimuli | Difference | Standard error | p-value | Adjusted p-value |
| --- | --- | --- | --- | --- | --- | --- | --- |
| Occipital cortex | 50-65 | -0.82 | 6.4 | 7.23 | 1.47 | 4.6 · 10^-5^ | 0.00037 |
|  | 65-80 | 1.46 | 5.4 | 3.94 | 1.49 | 0.014 | 0.098 |
| Thalamus | 50-65 | 2.20 | 4.94 | 2.73 | 2.12 | 0.210 | 0.630 |
|  | 65-80 | -0.40 | 3.33 | 3.73 | 2.15 | 0.094 | 0.470 |
| Caudate | 50-65 | -0.74 | 1.58 | 2.32 | 1.49 | 0.130 | 0.520 |
|  | 65-80 | -1.66 | -2.80 | -1.14 | 1.53 | 0.460 | 0.630 |
| Anterior cingulate cortex | 50-65 | -1.44 | 1.31 | 2.75 | 1.38 | 0.055 | 0.330 |
|  | 65-80 | -1.58 | -3.22 | -1.63 | 1.40 | 0.252 | 0.630 |


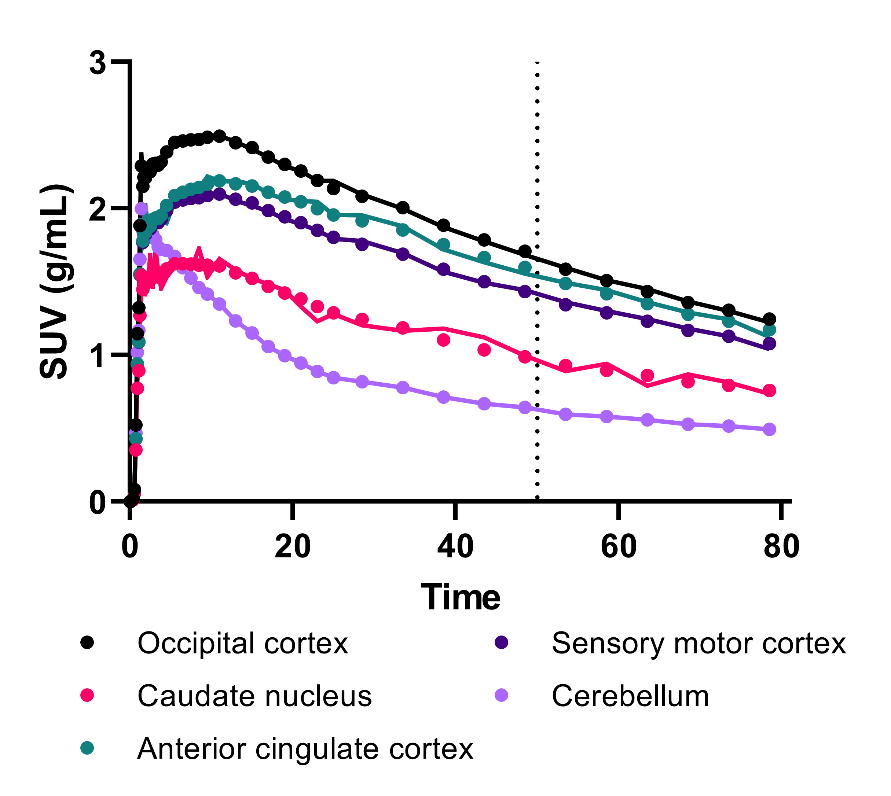


**Supplementary figure 3.** A representative [^11^C]AZ10419369 time-activity curve from a stimulus session for five different regions of interest. Symbols represent measured radioactivity normalized to injected dose and weight of the participant yielding standardized uptake value (SUV). Lines represent model fit of the extended simplified reference tissue model. The vertical dotted line represents the onset of the visual stimuli.
